# Construction and validation of prognostic nomogram for metaplastic breast cancer

**DOI:** 10.17305/bjbms.2021.5911

**Published:** 2021-06-10

**Authors:** Yongfeng Li, Daobao Chen, Haojun Xuan, Mihnea P. Dragomir, George A. Calin, Xuli Meng, Meng Chen, Hongchuan Jin

**Affiliations:** 1 Department of Cancer Biology, Key Lab of Zhejiang Biotherapy, Sir Run Run Shaw Hospital, Zhejiang University School of Medicine, Hangzhou, Zhejiang, China; 2 Department of General Surgery, Breast Surgery, Zhejiang Provincial People’s Hospital, Affiliated People’s Hospital, Hangzhou Medical college, Hangzhou, Zhejiang, China; 3 Department of Breast Surgery, Institute of Cancer Research and Basic Medical Sciences of Chinese Academy of Sciences (Zhejiang Cancer Hospital), Hangzhou, Zhejiang, China; 4 Department of Experimental Therapeutics, The University of Texas MD Anderson Cancer Center, Houston, Texas, United States; 5 Department of Pathology, Charité-Universitätsmedizin Berlin, Corporate Member of Freie Universität Berlin, Humboldt-Universität zu Berlin and Berlin Institute of Health, Berlin, Germany; 6 Center for RNA Interference and Non-Coding RNAs, The University of Texas MD Anderson Cancer Center, Houston, Texas, United States

**Keywords:** Metaplastic breast cancer, nomogram, overall survival, cancer-specific survival

## Abstract

In this study, we aimed to develop nomogram models for predicting the overall survival (OS) and cancer-specific survival (CSS) of patients with metaplastic breast carcinoma (MBC). Data of patients diagnosed with MBC from 1973 to 2015 were collected from the Surveillance, Epidemiology, and End Results database. Univariate and multivariate Cox analyses were performed to identify independent prognostic factors for OS and CSS of MBC patients. The obtained prognostic variables were combined to construct nomogram models for predicting OS and CSS in patients with MBC. Model performance was evaluated using concordance index (C-index) and calibration plots. Data from 1125 patients were collected and divided into a training (750) and a validation (375) cohort. The multivariate Cox model identified age, TNM stage, tumor size, and radiotherapy as independent covariates associated with OS and CSS. The nomogram constructed based on these covariates demonstrated excellent accuracy in estimating 3-, and 5-year OS and CSS, with a C-index of 0.769 (95% confidence interval [CI], 0.731-0.808) for OS and 0.761 (95% CI, 0.713-0.809) for CSS in the training cohort. In the validation cohort, the nomogram-predicted C-index was 0.738 (95% CI, 0.676-0.800) for OS and 0.747 (95% CI, 0.667-0.827) for CSS. All calibration curves exhibited good consistency between predicted and actual survival. The nomogram models established in this study may enhance the accuracy of prognosis prediction and therefore may improve individualized assessment of survival risks and enable constructive therapeutic suggestions.

## INTRODUCTION

Metaplastic breast carcinoma (MBC) is a relatively rare form of breast cancer, accounting for 0.2-5% of all breast cancers [1], with worse clinical outcomes and resistance to neoadjuvant systemic chemotherapies [2]. The incidence of MBC has been increasing since it was recognized as a distinct pathological diagnosis in 2000 [3]. Histologically, MBC is classified into several subtypes, including spindle, squamous, chondroid, osseous, and/or rhabdomyoid MBC [4]. MBC commonly shows a triple negative breast cancer phenotype, due to the lack of expression of the estrogen receptor (ER), progesterone receptor (PR), and human epidermal growth factor receptor-2 (HER2) [5], and is managed with surgical resection in combination with radiotherapy and chemotherapy [2]. However, only radiotherapy showed an improvement in overall survival (OS) of MBC patients [6,7]. Compared with invasive ductal carcinoma, the 5-year survival rate for MBC remains poor due to its rapid tumor growth rate and chemoresistance [8,9]. The Surveillance, Epidemiology, and End Results (SEER) database is an annually updated and population-based database in the USA, covering about 30% of the US population. It has become a distinctive resource to investigate special and rare malignancies, such as MBC, by taking advantage of its wide range of data on cancer.

Nomograms have been proposed as a novel and dependable tool to incorporate demographic and clinicopathological factors for accurate prognostic prediction of many cancers [10,11]. They have been generated from regression analysis and have shown to be comparable to the established tumor-node-metastasis (TNM) staging systems. At present, to the best of our knowledge, there are no available nomograms for predicting survival of the MBC patients. Two nomograms were developed for MBC, one for analyzing the role of chemotherapy in MBC [12] and second for the preoperative prediction of lymph node metastasis (LNM) in MBC patients [13]. Both studies show the utility of using nomograms for the analysis of MBC in the SEER database. Herein, we aimed to establish a novel nomogram for forecasting individualized survival of MBC depending on the personalized demographic, pathological, and therapeutic information from the SEER database.

## MATERIALS AND METHODS

### Patient population

**FIGURE 1:**
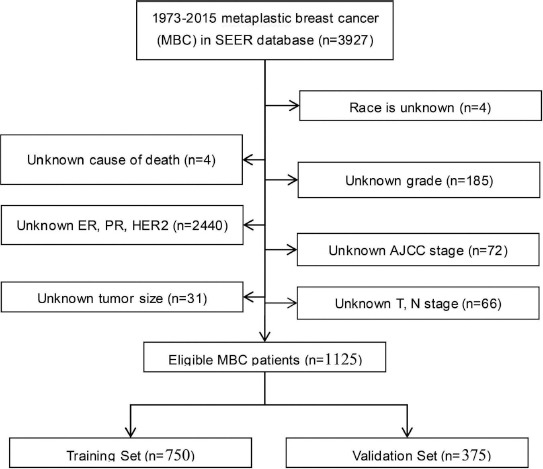
The flow chart of patient selection.

Data of MBC patients diagnosed between 1973 and 2015 were obtained from the SEER program of the National Cancer Institute (USA). Variables of interest for each case included age at diagnosis, race, grade, histology, status of ER, PR and HER2, American Joint Committee on Cancer (AJCC) tumor stage (7^th^ Edition), T stage, N stage, exact tumor size, and treatment information (including chemotherapy and adjuvant radiotherapy) were obtained from the database. The following SEER ICD-0-3 codes, including 8052, 8070-8072, 8074, 8560, 8571, 8572, 8575, and 8980, were adopted to identify cases of MBC [12]. Patients with missing demographic, pathological, or survival data were excluded from the study. Figure 1 illustrates the detailed flow diagram for patient inclusion. The current study is in accordance with the 1964 Helsinki Declaration and its later amendments, and was conducted in accordance with the ethical standards of the research committees of Zhejiang Cancer hospital.

### Threshold selection for continuous variables

**FIGURE 2:**
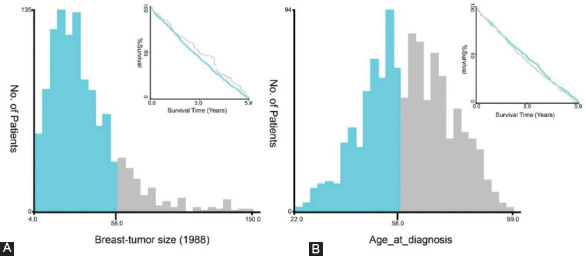
The X-tile analysis was implemented for the MBC patient cohort to ascertain the optimal threshold for (A) tumor size, and (B) age. The figure shows that the optimal cutoff point for tumor size and age for MBC patients is 58 mm (A) and 58-year-old (B).

The optimal cutoff values for the tumor size and age were calculated using X-tile software. The analysis showed that the optimal cutoff values for the tumor size and age are 58 mm and 58-years-old, respectively (Figure 2). This data were used to divide the cohorts into two groups based on the optimal cut-off value.

### Nomogram construction and conformation

Patients were randomly divided into the training set and validation set at a ratio of 2:1 using random split-sample method [14]. Univariate and multivariate analyses were carried out by employing the Cox proportional hazard regression models to determine the hazard ratio along with corresponding 95% confidence interval (CI) for all possible risk factors. All independent risk factors were identified by the forward stepwise selection method using the multivariate Cox proportional hazards models. The nomogram was established by combining all independent risk factors for the prediction of the 3-year and 5-year OS and cancer-specific survival (CSS) using the “RMS” R package (cran.rproject.org/web/packages/rms). The Harrell’s concordance index (C-index) was used to access the discrimination. Calibration curves were applied to estimate the consistency between the actual prognosis and the nomogram-predicted survival probability of the model.

### Ethical statement

Data used in the present study were obtained freely from the SEER database, which is a public research resource. Therefore, ethical approval for the study was exempted by the institutional review board.

### Statistical analysis

IBM SPSS statistics 22 software (SPSS Inc., Chicago, IL, USA) was used to conduct statistical analysis. R software v 3.6.1 (http://www.r-project.org) was adopted to construct nomograms based on the multivariate results and the “RMS” package was used to develop survival models. A two-tailed *p* < 0.05 was considered statistically significant.

## RESULTS

### Patients’ features

We enrolled in this study 1125 patients with MBC. The demographic and clinical characteristics of the included patients are presented in Table 1. Of these patients, 637 (56.62%) were diagnosed with MBC at the age of 58 years or older. We selected the 58-years-old threshold by using x-tile software to obtain an optimal cutoff value for continuous variables. The majority of the patients 862 (76.62%) were white. Using the same x-tile software to obtain an optimal cut-value for tumor size, the patients were divided into two groups, including 967 patients with tumor size <58 mm, and 161 patients with tumor size >58 mm. Regarding the degree of cancer cell differentiation, Grade 3 was the most common type, 904 patients (80.36%). Regarding the hormone receptors and HER2 status, 880 (78.22%) were ER negative, 975 (86.67%) were PR negative, and 1061 (94.31%) were HER2 negative. According to the AJCC7 system, Stage II was the dominant one 695 (61.78%), followed by Stage I 269 (23.91%) and Stage III 161 (14.31%). Most patients were diagnosed with T2 stage 583 (51.82%). The majority of the patients, 880 (78.22%) were diagnosed as N0 stage. Regarding the therapy regime, more than half of patients 747 (66.40%) had undergone chemotherapy, while 529 (47.02%) patients had undergone radiotherapy. For validation proposes, the entire patient cohort was divided into the training cohort (750) and validation cohort (375).

**Table 1:**
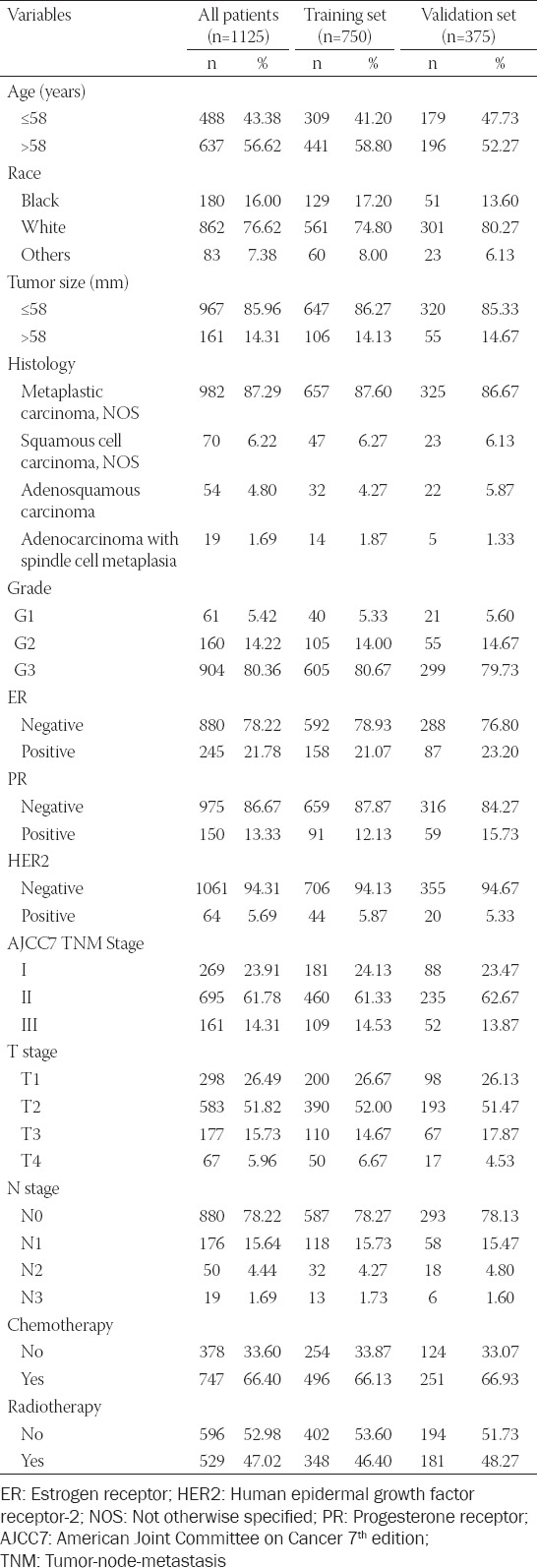
Characteristics of the training and validation cohorts

### Prognostic factors of OS and CSS

According to the univariate analysis performed among the training cohort, eight variables, including age (*p* < 0.001), tumor size (*p* < 0.001), AJCC TNM stage (*p* < 0.001), T stage (*p* < 0.001), N stage (*p* < 0.001), chemotherapy (*p* < 0.001), and radiotherapy (*p* < 0.001), were significantly associated with OS in patients with MBC (Table 2). These variables, except for age and chemotherapy, were also found to be significantly associated with CSS in the univariate analysis (Table 3). Next, multivariate analysis indicated that age (*p* = 0.001), AJCC TNM stage (*p* = 0.001), tumor size (*p* < 0.001), and radiotherapy (*p* = 0.001) are independent prognostic factors of OS of patients with MBC (Table 2). Moreover, age (*p* = 0.022), AJCC TNM stage (*p* = 0.001), tumor size (*p* < 0.01), and radiotherapy (*p* < 0.009) were also identified as independent prognostic factors of CSS of MBC patients in the multivariate analysis (Table 3).

**Table 2:**
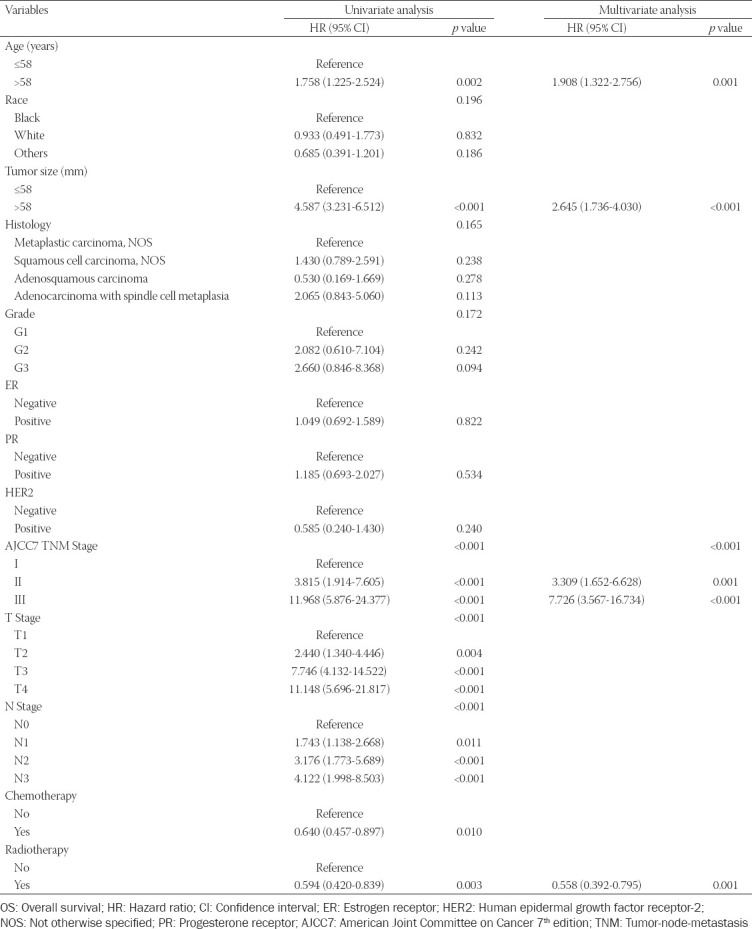
Univariate and multivariate analyses of variables associated with OS

**Table 3:**
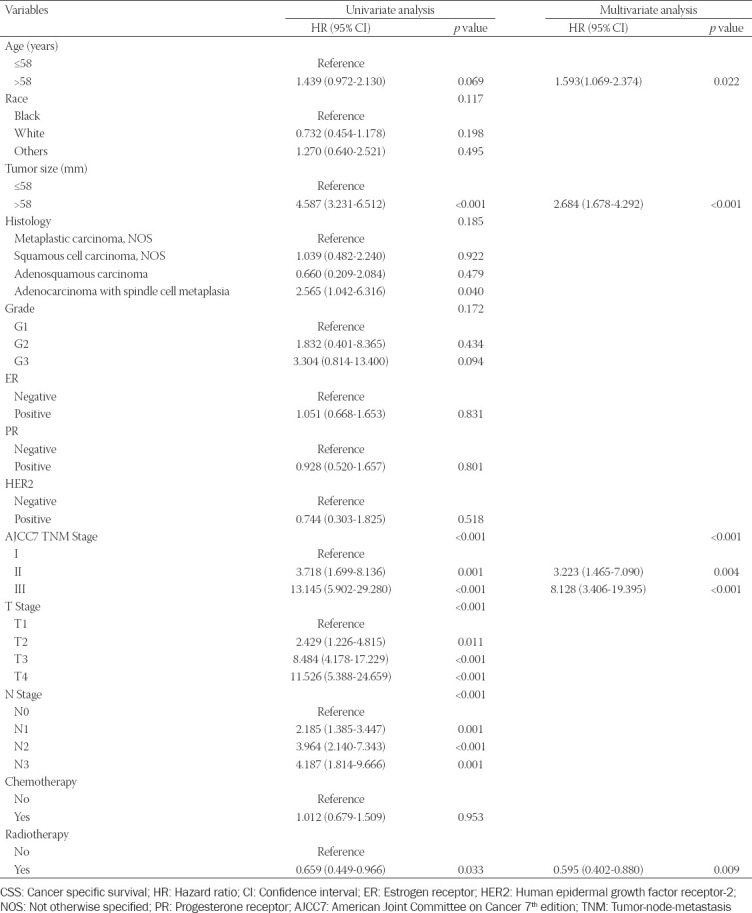
Univariate and multivariate analyses of variables associated with CSS

### Construction and validation of OS and CSS nomograms

**FIGURE 3:**
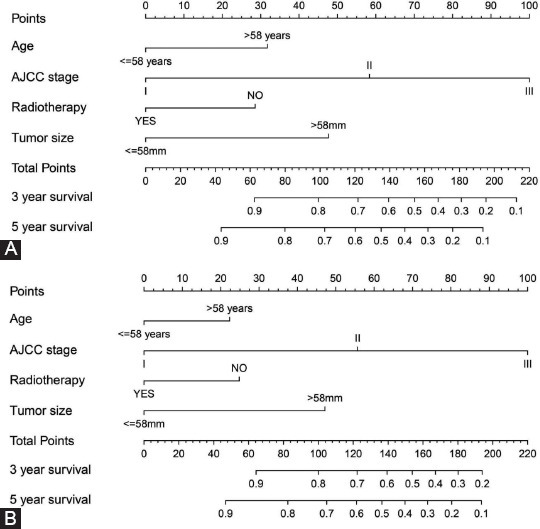
Nomograms for predicting the 3-, and 5-year (A) overall survival and (B) cancer-specific survival of MBC.

According to the results of the multivariate analysis, all independent prognostic factors in the training set were incorporated to create nomograms for estimating the 3- and 5- year OS and CSS of MBC patients. In Figure 3a and b, we presented the prediction of the 3- and 5-year OS and CSS in the nomograms, respectively.

**FIGURE 4:**
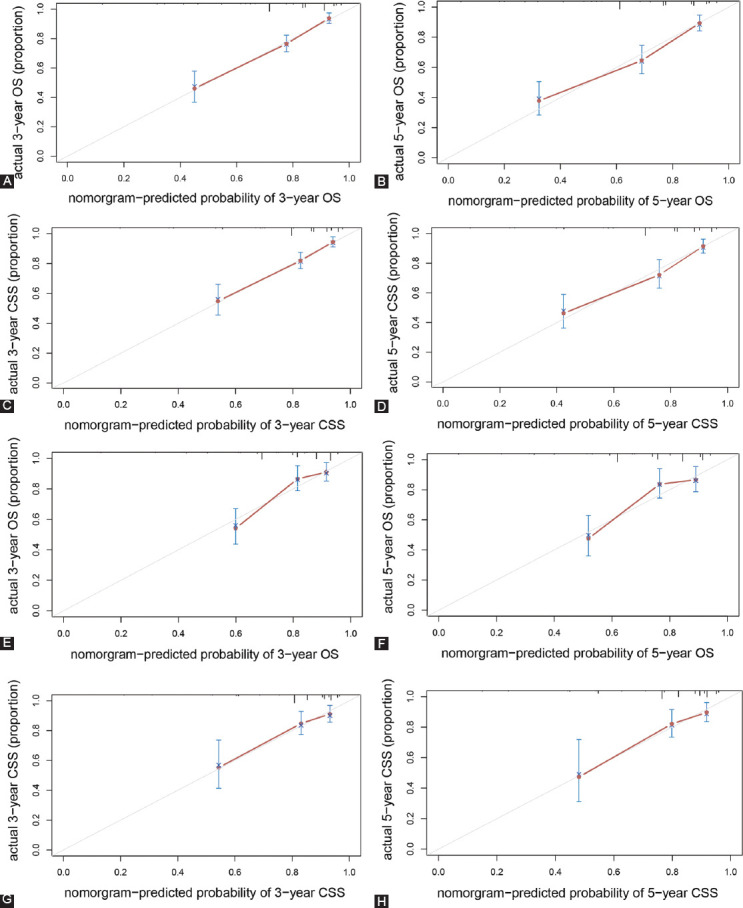
The calibration curves for predicting the survival of MBC patients. The overall survival (A and B) and cancer-specific survival (C and D) in the training cohort at 3 and 5 years after diagnosis, and the overall survival (E and F) and cancer-specific survival (G and H) in the validation cohort at 3 and 5 years after diagnosis.

The validation of the results showed sufficient accuracy in forecasting the prognosis of MBC in both sets. The C-index of the nomogram for OS and CSS was 0.769 (95% CI = 0.731-0.808) and 0.761 (95% CI = 0.713-0.809) in the training set (Table 4). The C-index calculated from the validation set was 0.738 (95% CI = 0.676-0.800) for OS and 0.747 (95% CI = 0.667-0.827) for CSS, respectively. The calibration plots showed good coordination between prediction by nomogram models and observed outcomes in the 3- and 5-year OS and CSS of patients with MBC in both training and validation cohort (Figure 4).

**TABLE 4:**
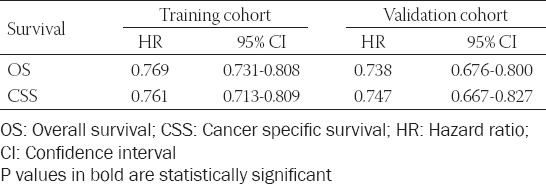
The C-index of nomogram for OS and CSS in patients with MBC

## DISCUSSION

MBC is a heterogenous subtype of breast cancer, which is relatively rare in everyday clinical practice. Despite several studies that found risk factors related to the clinical outcomes of MBC patients [15-17], there has been no attempt to construct a nomogram based on the various risk factors to predict the survival of MBC. Wright et al. [18] reported that for patients with positive or negative hormone receptors, there was no significant difference in the 5-year survival rate of MBC, which indicates that the status of hormone receptors may not be considered as a prognostic factor of MBC. In addition, a previous study demonstrated that the molecular subtype of MBC could be an independent predictor [3]. Several studies have revealed that the prognosis of MBC patients with larger tumor and LNM is generally poor [15,19]. In recent years, some studies have also focused on the relationship between gene signatures and prognosis of MBC patients, such as high expression of RPL39 [17] and the mutation of the colony stimulating factor 1 receptor [20], both of which being associated with poor prognosis. Single prognostic factors play a limited role in predicting individual survival probability. Nomograms are mathematical models for predicting cancer risk, and therapeutic outcomes and have become a popular clinical decision aid tool [21-23]. It has been revealed that nomograms show more excellent prediction precision and prognostic value in diverse malignancies than the AJCC TNM classification system [24,25]. In this study, we found that several clinicopathological characteristics were independent prognostic factors for OS and CSS of MBC patients, including age, AJCC TNM classification system, tumor size, and radiotherapy. The nomograms established in this study showed favorable discrimination and calibration for 3-year and 5-year OS and CSS of MBC patients, offering a more accurate, and personalized clinical tool for prognosis evaluation of MBC patients.

Prognostic studies have given variable results regarding factors associated with prognosis and survival of cancer patients [26-36]. In the present study, we critically evaluated the prognostic value of various factors based on a large sample of cases of the MBC recorded from the SEER database. The clinical significance of age, TNM stage, tumor size, and radiotherapy in MBC patients was highlighted in the nomogram models. Our result demonstrated that patients over 58 years show unfavorable survival with poor OS and CSS. Old MBC patients generally have a higher-risk histological subtype [37], which has been considered as an independent risk factor and may eventually result in lower survival [38-40]. Although the majority of patients (66.4%) from our cohort adopted chemotherapy, it had no significant improvement in OS and CSS. This may result from the minimal response to chemotherapy in MBC [27,31,33,41,42]. However, it is difficult to draw conclusions from our data given the lack of details regarding the ­chemotherapy (type, timing, etc.). the previous studies have concluded that radiotherapy was able to improve the survival of patients with MBC [8,28,38], and our data also demonstrated that radiotherapy is an independent prognostic factor, being associated with survival probability of patients with MBC [9,34,43]. Moreover, radiotherapy has shown to reduce the risk of local recurrence [44]. The addition of radiotherapy can reduce residual lesions in the surgical area or regional lymph nodes, as well as reduce local recurrence, and distant metastasis [45]. These may contribute to its independent effect on CSS of MBC patients. T stage represents the tumor size, and our results demonstrated that T4 had an impact on OS and CSS in MBC patients, which is in line with previous population-based study of MBC [9]. LNM has been identified as a key prognostic indicator for a variety of malignancies, and the number of LNM is a key element of the TNM-staging. The previous studies reported that lymph node status was significantly correlated with survival endpoints in patients with MBC [46,47]. In the present study, although a higher T and N stage predicted worse OS and CSS, they were not independent prognostic factors for OS or CSS. This may be due to the integration of T and N stage information in the AJCC stage.

There were several potential limitations in this study. First, retrospective data retrieved from the same database was used for the generation and validation of the nomogram models, which may lead to the risk of potential selection bias. Therefore, it would be more reliable to validate the nomograms in other clinical cohorts. Second, in this study, we only included the OS and CSS. The assessment of recurrence risk is considered as a more meaningful endpoint than OS or CSS, but is unavailable in the SEER database. Moreover, several other crucial prognostic factors, such as RET mutation status and calcitonin doubling times, were also unavailable in the SEER database.

## CONCLUSION

In the present study, age, AJCC stage, tumor size, and radiotherapy were identified as independent prognostic factors for OS and CSS of MBC. We successfully established and validated nomograms constructed using these independent prognostic factors that reliably predict the 3- and 5-year OS and CSS of MBC patients. These nomograms could assist clinicians to estimate the aggressiveness of the tumor and make individualized decisions.
